# Rebuttal letter of the “comment on “contrastive pre-training and 3D convolution neural network for RNA and small molecule binding affinity prediction” by Sun and Gao”

**DOI:** 10.1093/bioinformatics/btaf164

**Published:** 2025-07-12

**Authors:** Saisai Sun

**Affiliations:** School of Computer Science and Technology, Xidian University, Xi’an, Shaanxi 710071, China

Dear Editor,

Thank you for providing us with the opportunity to clarify and address the questions raised in the comment letter. We appreciate the thoughtful comments and the opportunity for a detailed discussion about our research. In the following sections, we have provided detailed responses to each of the questions posed. Our aim is to offer comprehensive explanations and to elucidate any aspects of our work that may have prompted further inquiry. We hope that our responses will address the concerns effectively and enhance the understanding of our research.

## 1 Presence of DNA-ligand complex structures from PDBbind among the training, validation, and test datasets

There are several reasons for including DNA-ligand complex structures from PDBbind (Wang *et al.* 2004) in our training, validation, and test datasets. Firstly, the PDBbind dataset is relatively small, making it impractical to exclude the DNA complexes entirely. Additionally, we have separated the double-stranded DNA into single chains, treating these as the binding targets. This approach aligns with the structure of some RNA single chains, such as PDB structure of 2f4s. Moreover, research has shown that RNA can adopt a wide conformational range between the canonical A- and B-forms at the localized single-strand domain (SSD) level, including many B-form-like conformations. This occurs through C2'-endo ribose conformations in one or both nucleotides and B-form-like neighboring base stacking patterns (Sedova and Banavali 2016).

## 2 MinMax transformation of output variable (pKd) affects generalization of model to unseen data

Firstly, translations (subtracting a constant) and scaling (multiplying by a constant) do not affect correlation. Since MinMax scaling is simply a combination of translation and scaling (without any shear), it does not impact cross-correlation. Therefore, the MinMax transformation cannot artificially inflate Pearson correlation coefficients (PCCs). Regarding the case discussed by Krishnan *et al.* the reported binding affinity of the theophylline aptamer to caffeine, which is 3500 μM (Jenison, *et al.* 1994; Menichelli, *et al.* 2022), may not correspond accurately to the PDB structure 1EHT, deposited by Zimmermann *et al.* in 1997 (Zimmermann, *et al.* 1997).

## 3 Inability of RLaffinity model to generalize to unseen RNA–ligand complexes

The results presented in Table 1 of the comment letter reflect a performance similar to that described in the RLaffinity manuscript (Sun and Gao 2024), which reported a PCC of approximately 0.56 and a Spearman’s rank correlation coefficient (SPCC) of around 0.54.

**Table 1.** The results from different methods on the benchmark test set.

Methods	PCC	SPCC	RMSE	MAE
Vina	−0.386	−0.389	0.277	0.257
RF-score	0.445	0.364	0.152	0.129
RSAPred	0.399	0.221	2.886	2.205
3DCNN	0.466	0.408	0.165	0.129
RLaffinity	0.559	0.540	0.152	0.119

## 4 Prediction performance is not better than existing methods

Table 1 demonstrates that RSAPred can achieve a PCC of 0.90 and a SPCC of 0.75 on the seven riboswitch-ligand pairs, using the RSAPred riboswitch model for predictions. According to the RSAPred paper (Krishnan, *et al.* 2024), RSAPred exhibited the best performance on riboswitches compared to other types of RNA-ligand pairs. In contrast, RLaffinity was trained on all types of RNA–ligand pairs, not specifically on riboswitch–ligand pairs, which may explain its lower performance in this context. Additionally, the mean absolute error values of RLaffinity and RSAPred are comparable. However, a comparison across all RNA–ligand pair types shows that RSAPred is less effective on type-insensitive RNA–ligand pairs (Sun and Gao 2024).

## 5 Incorrect comparison with existing methods

Firstly, Vina and RF-score are also capable of predicting RNA-small molecule interactions. Due to the limited availability of RNA-small molecule binding affinity prediction methods, RLaffinity used Vina and RF-score as baseline methods for comparison, providing additional context for evaluating RLaffinity’s performance. As mentioned earlier, linear (Minmax) normalization does not affect the PCCs between predicted and actual values. RLaffinity is specifically designed for type-insensitive RNA-small molecule binding affinity prediction, which is why the comparison with RSAPred (Krishnan *et al.* 2024) did not account for RNA types. This approach also allows for evaluating RSAPred’s performance on type-unknown RNAs.
